# Targeted metabolomics of organic and amino acids in giraffe milk during mid- to late-lactation

**DOI:** 10.1007/s11306-026-02455-z

**Published:** 2026-05-16

**Authors:** G. Osthoff, S. Mason, E. Davoren, F. Deacon

**Affiliations:** 1 Department of Microbiology and Biochemistry, University of the Free State, Bloemfontein, South Africa; 2 Biomedical and Molecular Metabolism Research (BioMMet), Faculty of Natural and Agricultural Sciences, North-West University, Potchefstroom, South Africa; 3 Centre for Human Metabolomics, Desmond Tutu School of Medicine, Faculty of Health Science, North-West University, Potchefstroom, South Africa; 4 Department of Animal, Wildlife and Grassland Sciences, University of the Free State, Bloemfontein, 9300 South Africa

**Keywords:** Giraffe, Ruminant, Milk, Lactation, Mammary involution, Organic acids, Amino acids, Targeted metabolomics

## Abstract

**Background:**

At the end of lactation (mammary involution), dynamic changes in milk components occur in all mammals. The time to reach complete cessation varies among taxa and species. The involution of cows, sheep, and goats (Bovidae) has been reported, but limited information is available on giraffes.

**Objectives:**

Characterise organic acids and amino acids in the milk of giraffes at involution.

**Methods:**

Milk was obtained from five giraffes. A LC-MS/MS metabolomics approach was followed, and statistical analysis of the data was done using MetaboAnalyst 6.0.

**Results:**

There were 38 organic acids and 45 amino acids measured in the giraffe milk. The organic acids indicate a decrease in Krebs cycle intermediates. Lower citrate levels are associated with lower lactose levels, indicating reduced osmotic regulation. Lower uracil and orotic acid indicate decreased pyrimidine synthesis and eventual nucleotide synthesis. Increased amino acid content is not devoted to protein synthesis, but to other functions, specifically as antioxidants, redox buffering, and cytoprotection. Increased histidine, serine and methionine promote protein degradation and one-carbon metabolism. Lysine catabolites lead to decreased levels of energy metabolites and to stress adaptation. Aromatic amino acids modulate the supply of immune and neuroactive metabolites.

**Conclusions:**

During involution, the regulation of organic acids suggests reduced Krebs cycle activity, indicating a transition from high biosynthetic to catabolic activity. Likewise, amino acids have other functions, specifically antioxidant, redox-buffering, and cytoprotection.

**Supplementary Information:**

The online version contains supplementary material available at 10.1007/s11306-026-02455-z.

## Introduction

In mammals, lactation is an essential process for the nourishment and development of the offspring. This process is divided into four stages: mammogenesis (development of the mammary gland), lactogenesis (initiation of milk secretion), galactopoiesis (continuous milk secretion), and involution (cessation of lactation) (Rezaei et al., 2016). The end of lactation, also known as the dry-out period or involution, is the transition from the lactating to the non-lactating state. When offspring are naturally weaned over time, this transition may be gradual. It could be initiated by the sudden cessation of milk removal, as is practised in livestock management (Hurley, 1989). Initiated involution has been reported in detail in commercially exploited mammals, mainly the cow (Hurley, 1987; Boggs et al., 2015; Peaker & Wilde, 1996), sheep (Petridis & Fthenakis, 2019), and goat (Shamay et al., 2002), as well as in humans (Hartmann & Kulski, 1978). During this period, milk production is shut down as milk gland cells are remodelled into a non-lactating state (Hurley, 1989). This period is properly managed in the milk production industry due to the udder’s susceptibility to infection and the clinical mastitis caused mainly by *Staphylococcus aureus* (Andreotti et al., 2014; Renna et al., 2019). Preventive measures include providing clean housing and bedding, adapting nutrition, and supplementing with vitamins and minerals (McMullen et al., 2021). A well-managed healthy state of dry-out and dry period also assures optimal functioning of the milk gland during the next lactation (Odensten et al., 2007; Hurley & Loor, 2011).

Once nursing is stopped, the accumulated milk in the udder lumen and the epithelia stimulates a shutdown process that takes approximately seven days. The lactose content decreases within 36 h of stopping nursing and reaches its lowest level within seven days. The fat content remains relatively constant for three days, then decreases after seven days. Protein content peaks at three days and then decreases (Hurley, 1987; Shamay et al., 2002). Simultaneously, the sodium (Na) content increases and the potassium (K) content decreases after 12 h (Shamay et al., 2002). The increase in protein content includes lysozyme, lactoferrin, serum albumin, immunoglobulins, complement proteins, and cathelicidins, while caseins, α-lactalbumin and β-lactoglobulin decrease (Hartmann & Kulski, 1978; Boggs et al., 2015). Pathogen-recognition proteins, such as immunoglobulins, lactoferrin, complement proteins, and cathelicidins, form a barrier against mastitis-causing pathogens (Boggs et al., 2015). Within 12 h, the high protein content also stimulates increased activation of plasminogen to plasmin. Peptides, mainly originating from the α- and β-caseins, are then produced by plasmin hydrolysis. The resulting casein-derived phosphoproteins (Boggs et al., 2015) and β-casein fragment β-CN 1–28 (Silanikove et al., 2000) inhibit K-secretion by closing K-channels and disrupting the tight junctions between milk-secreting cells to induce dry-out (Nguyen & Neville, 1998). These proteins and peptides form a keratin plug at the teat orifice, thereby preventing pathogen access. Simultaneously, a 7,600 Dalton protein, which is a feedback inhibitor of lactation, reduces the rate of lactation by inhibiting the transfer of proteins through the Golgi apparatus in the mammary epithelial cells (Peaker & Wild, 1996). The process of mammary gland involution is controlled by sex steroid hormones, which decrease prolactin and somatotropin secretion, thereby inhibiting the release of galactopoietic hormones (Lamote et al., 2004).

The human breast milk metabolome has been studied in some detail (Dessì et al. 2018; Nolan et al. 2021; Li et al. 2022a). Far less is known of the milk metabolome of animals, and most reports focus on domesticated animals such as the cow (*Bos taurus*) (Scano et al., 2014; Tenori et al., 2018; Settachaimongkon et al., 2021), goat (*Capra hircus*) (Cabrera et al., 2023), sheep (*Ovis aries*) (Cabrera et al., 2023), donkey (*Equus asinus*) (Li et al., 2020; Laus et al., 2023; Garhwal et al., 2023; Yang et al., 2024), and Bactrian camel (*Camelus bactrianus*) (Li et al. 2022b). Information from milk metabolomics is applied to dairy herd authenticity (Tenori et al., 2018), animal nutrition (Xu et al., 2020), and food quality and traceability (Rocchetti & O’Callaghan, 2021). Very little information is available for other species (Osthoff et al., 2023, 2024, Osthoff et al. 2025a). In the cited research reports, some focus was placed on the metabolomic changes during early lactation from colostrum to mature milk. Collectively, it is difficult to compare the data and draw conclusions because the milk samples were not collected at the same intervals throughout lactation, and the analytical methods differed. Some were carried out by water extraction and ^1^H-NMR (Settachaimongkon et al., 2021; Garhwal et al., 2023) and others by chromatography and MS techniques (Li et al., 2020; Yang et al., 2024). Concurrently, there was no consensus on which metabolic pathways were up- or down-regulated. Nevertheless, in general, the amounts of amino acids and their derivatives, organic acids, and lipid metabolites decreased over lactation, while carbohydrates and their derivatives increased.

We recently investigated the milk metabolome and its changes during mammary gland involution in the giraffe (*Giraffa camelopardalis*), the largest ruminant in Africa. It falls under the family Giraffidae and the suborder Ruminantia. Its milk composition has been studied in detail (Hall-Martin et al., 1977; Osthoff et al., 2017) and found to differ from that of other ruminants, such as Bovini, Tragelaphini, and Alcelaphinae, particularly with a high protein (4.9%), fat (7.94%) and unsaturated fatty acid (25% of the total fatty acids) content and a fatty acid composition. The milk metabolome of the giraffe was also shown to differ between species or taxa (Osthoff et al. 2025a), and the changes in proximate composition of giraffe milk from mid to late lactation were described (Osthoff et al. 2025b).

Regarding changes in the metabolome during milk gland involution, as determined by ^1^H-NMR, we showed that acids increased, while organic acids, lipid metabolites, and carbohydrates and their derivatives decreased (Osthoff et al., 2026). This indicated that amino acid and protein synthesis were upregulated, whereas lipid and carbohydrate synthesis were downregulated. Energy-producing amino acids and citric acid cycle intermediates decreased, suggesting reduced availability of energy metabolites. Since organic acids, amino acids, and their derivatives played a major role in the metabolomic changes during giraffe mammary involution, we deemed it appropriate to conduct an in-depth study of these components using targeted LC-MS/MS metabolomics.

## Materials and methods

### Animal handling and sampling procedures

The keeping and welfare of the giraffes under study were recently published in the same journal (Osthoff et al., 2026). Milk was obtained from five giraffes (Andri, Ayanda, Katherine, Marietjie, and Tiffany) at Amanzi Game Reserve in Brandfort, Free State Province, South Africa. All giraffes followed the same diet, a factor known to influence milk composition in domesticated mammals, specifically the fat content and fatty acid composition (Sánchez et al., 2006). The current study was part of fertility studies conducted between January and September 2024, during which the animals were tranquilised in accordance with routine ethical procedures (Deacon et al., 2022). A 100% success rate in immobilising free-ranging giraffes with zero mortalities or injuries was achieved. The preferred protocol used was Thianil (A-3080), administered via a dart gun at ≤ 40 m into the shoulder or rump for optimal absorption. Knockdowns were usually between 4 and 8 min. Once recumbent, an antagonist was administered immediately to prevent respiratory depression (Deacon et al., 2022). Each operation was supervised by at least two experienced wildlife veterinarians. Terrain, visibility, and environmental safety were critical factors in planning to ensure humane, controlled immobilisation. No information on parity was available for the animals. The age of the calves was estimated to be between six and eight months based on their size at the first encounter. It was concurrently estimated that the first sampling for this study was conducted at 8.9 months of lactation.

Investigations were initially conducted at weekly intervals but were later reduced as information on the animals’ condition became available. This resulted in sample heterogeneity that may limit statistical power and generalizability, which in turn may influence metabolite profiles. We and acknowledge this as a major limitation. Milk was drawn by palpation of the teats and sustained pressure exerted on the udder. Nitrile examination gloves (Lasec, Bloemfontein, South Africa) were worn during milking. Teats were milked out into sterile plastic cuvettes, producing 10–20 mL of milk per teat. Milk from the teats was collected separately, but the samples were pooled once it was established that all four quarters showed no discolouration or off-odours. Milk was cooled in an ice box to approximately 5 °C and transported to the laboratory within 4 h. Milk samples were then divided into fractions for chemical analysis, which were kept frozen until analysed. Milk serum was prepared for metabolomics analysis according to the methods described by Osthoff et al. (2026). Briefly, samples were filtered with Amicon Ultra 2 mL centrifugal units with 10 kDa membrane filters (Merck, Darmstadt, Germany). The filtrate was centrifuged at 12,000 x *g* for 5 min, and 600 µL was collected from the middle layer as ‘serum’. A pooled quality control (pQC) sample was created by aliquoting 50 µL from each milk serum sample.

### LC-MS/MS analysis of amino acids

All reagents and consumables — including mobile phases, internal standards, calibrators, quality control samples, 96-well plates, and the analytical column — were sourced from the ChromSystems MassChrom Amino Acid Analysis in Plasma/Serum kit (distributed by Separations, Johannesburg, South Africa). Milk serum samples, calibrators, and quality control samples (25 µL each) were pipetted into 1.5 mL Eppendorf tubes, followed by the addition of 50 µL isotope mix and 400 µL precipitation reagent. Samples were mixed for 30 s and centrifuged at 800 x *g* for 5 min. The supernatant was transferred into a 96-well plate. Samples (5 µL) were analysed on an Agilent Infinity 1290 binary pump paired with a 6470 MS/MS using positive electrospray ionisation. Gradient chromatography was conducted at 25 °C with an initial condition of 100% mobile phase A and a flow rate of 0.8 mL/min. The total analysis time was 20.5 min.

### LC-MS/MS analysis of organic acids

Milk serum samples were thawed to room temperature and homogenised via vortexing. Each sample (50 µL) was aliquoted into a microcentrifuge tube. To the tubes, an internal standard mix (methylmalonic acid-d3: 41.3 µM, hexanoylglycine-d2: 25 µM) and 200 µL of HPLC-grade water containing 0.1% formic acid were added, then vortexed to mix. A volume (200 µL) was transferred to 2 mL glass vials with inserts for analysis. Samples were analysed on an Agilent Infinity 1290 binary pump paired with an Agilent 6475 LC-MS/MS, using negative electrospray ionisation in MRM mode. Mobile phases consisted of (A) water and (B) acetonitrile, both containing 0.1% v/v formic acid (HPLC-grade, Burdick and Jackson). Chromatographic separation was performed on an Acquity UPLC HSS T3 column (1.8 μm, 2.1 × 100 mm) with an Acquity VanGuard Pre-column (2.1 × 5 mm; Waters, South Africa). The column and autosampler temperatures were maintained at 25 °C and 6 °C, respectively. The flow rate was set to 0.38 mL/min, with a 2 µL injection volume. The 28 min gradient was as follows: t = 0–2 min 100% A; t = 2–12 min 60% A; t = 12–24 min 5% A; t = 24–25 min 100% A; t = 25–28 min 100% A. To ensure analytical quality, blanks were randomised across batches, and pQC samples were injected at the beginning, middle, and end of the batch.

### LC-MS/MS analysis of glutathione

Reduced (GSH) and oxidised (GSSG) glutathione levels in milk samples were quantified by adapting the LC–MS/MS approach of Moore et al. (2013). Milk serum samples were treated with N-ethylmaleimide (NEM) to stabilise thiols, subjected to protein precipitation, and spiked with a NEM-treated stable-isotope (glutathione-[glycine-¹³C₂¹⁵N] trifluoroacetate; Sigma-Aldrich) prior to analysis. Chromatographic separation was employed using an Agilent ZORBAX RRHT Eclipse XDB-C18 column (1.8 μm, 4.6 × 50 mm) held at 25 °C. A 1 µL injection was delivered at a flow rate of 0.4 mL/min. Mobile phase A was water with 0.1% formic acid, and mobile phase B was acetonitrile with 0.1% formic acid. The gradient program and MRM transitions were performed according to the published protocol (Moore et al., 2013). Mass spectrometric source conditions were set to provide robust ionisation and included a drying gas temperature of 350 °C with a flow of 6 L/min, a sheath gas temperature of 200 °C and a flow of 10 L/min, a nebuliser pressure of 25 psi, a capillary voltage of 3000 V, and a nozzle voltage of 1000 V.

### Data analysis

Data acquisition and quantification were performed using Agilent MassHunter Data Acquisition and MassHunter Quantitative for QQQ software (version 10.0), respectively. Absolute quantification was achieved for amino acid analysis using a four-point calibration curve and isotopically labelled internal standards. In contrast, the organic acid analysis used a semi-quantitative (relative) approach. Peak areas of the analytes were normalised to a corresponding internal standard (either methylmalonic acid-d3 or hexanoylglycine-d2) and reported as response ratios, without the use of external calibration curves. This approach was applied due to the absence of a fully validated calibration model and the limited availability of isotopically labelled internal standards for the full organic acid panel.

### Statistical analysis

Statistical analyses were done using Excel and MetaboAnalyst 6.0 (www.metaboanalyst.ca/*).* Nonparametric (untransformed) data were used for univariate analyses, while log transformation and Pareto scaling were done to generate parametric data for multivariate analyses. Unsupervised principal component analysis (PCA) was done to visualise the data. For univariate analyses, analysis of variance (ANOVA) was used to compare more than two groups, and Student’s t-test was used to compare two groups, with statistical significance defined as an FDR *p* < 0.05. Due to the small sample size, Hedge’s effect size (ES; g-value), instead of Cohen’s d-value, was calculated using Excel to evaluate practical significance. A large effect (high practical significance) was determined as a g-value > 0.8. A strong linear correlation was defined as a correlation of determination (R^2^) > 0.6. Heatmaps (Pearson distance and Ward clustering) of the significant compounds were also generated. Linear regression plots were created in Excel to visualise the linear trends of metabolites.

### Metabolite analysis

Online metabolite databases (KEGG, HMDB, PathBank) and the literature were used to link the statistically significant metabolites to metabolic pathways. BioRender (www.biorender.com*)* was used to generate the metabolic pathways for discussion purposes.

## Results

All five giraffes studied were not sampled at the same intervals or across all lactation stages. The reason was that certain animals were excluded from work later in the study as information became available. Due to time constraints and efforts to reduce stress, data on milk collected between 8.9 and 9.6 months of lactation were thus centred at 9.4 months, and collections between 11.7 and 12.2 months were centred at 12 months. Milk from giraffe cows, Ayanda, Katherine, and Marietjie, at 15.1 months of lactation, had a light brownish colour. A last attempt to collect milk at approximately 16 months of lactation was unsuccessful because the giraffe cows were no longer lactating.

It should be noted that it is unclear whether the anaesthetic agent used, thianil, could alter the milk metabolite composition, as no literature is available.

### Metabolite profile

**Fig. 1:**
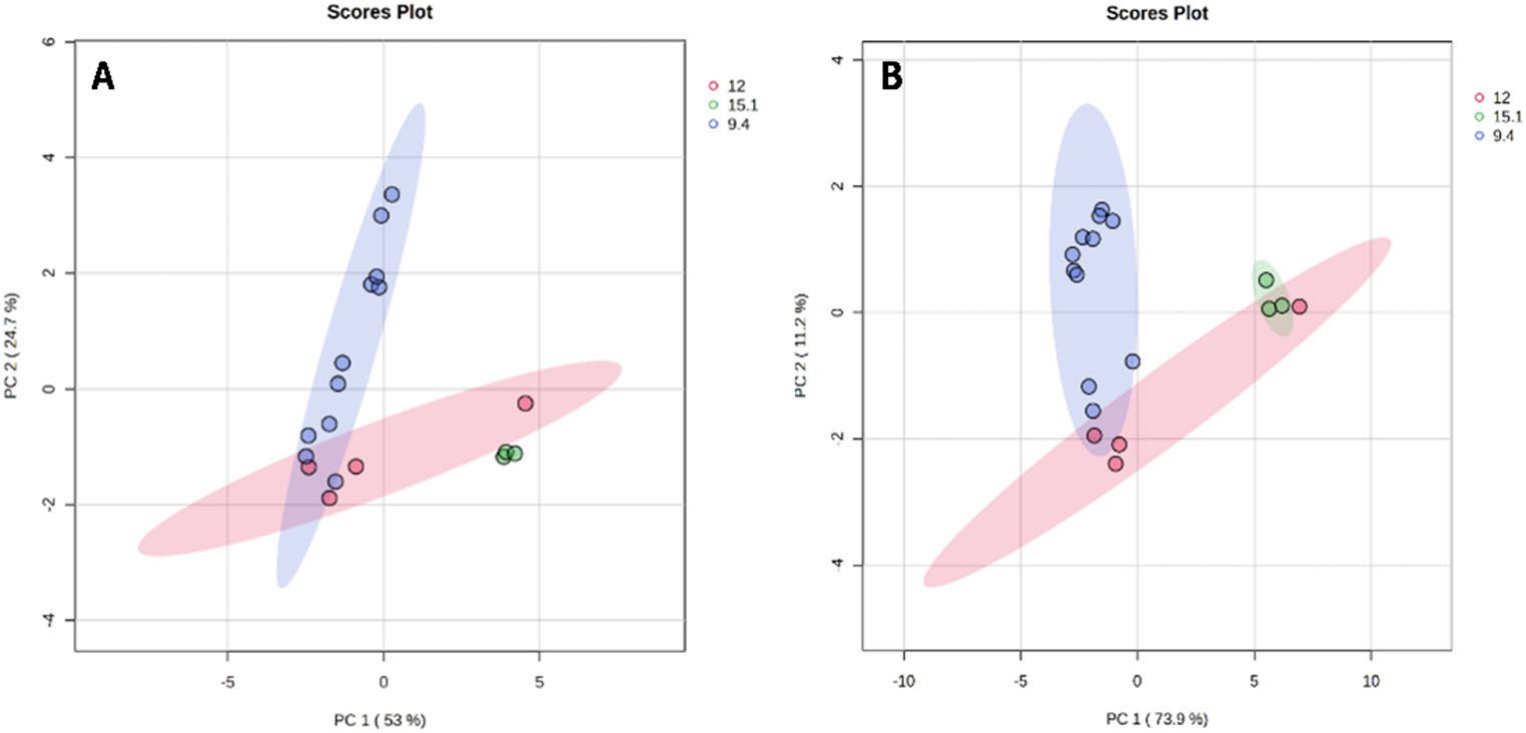
Multivariate statistical analysis (PCA) of organic acids (**A**) and amino acids with derivatives (**B**) in giraffe milk at 9.4 (blue), 12 (red), and 15.1 (green) months of lactation. The 9.4-month and 15.1-month clusters are separated from each other, indicating a change in metabolism between mid-lactation (9.4 months) and late-lactation (15.1 months). The 12-month group showed that some giraffes were still in mid-lactation, while others were either transitioning or already at end-stage lactation

**Fig. 2:**
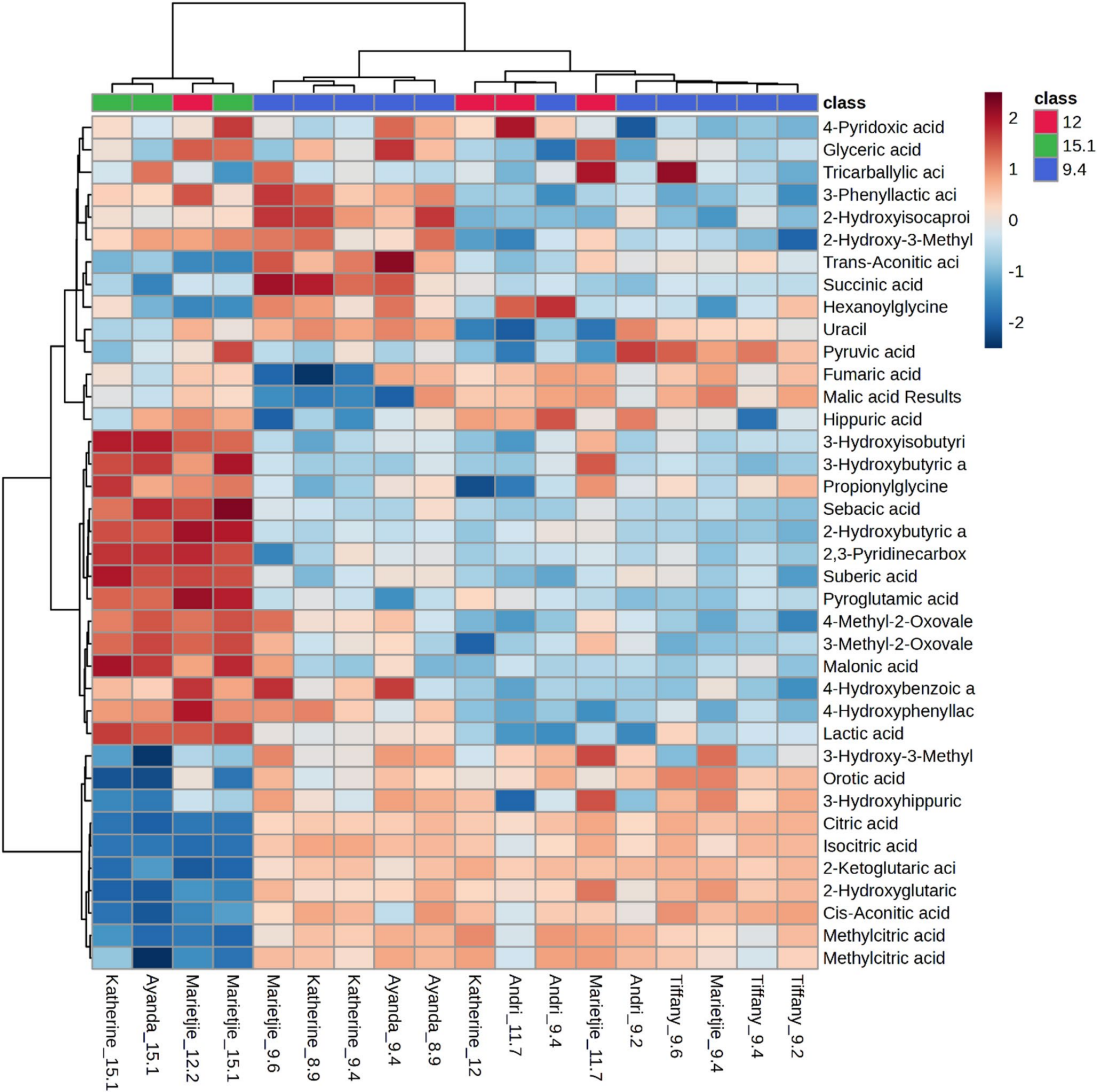
Heat-map visualisation and hierarchical clustering of organic acid profiles of giraffe milk at 9.4, 12, and 15.1 months of lactation. The dendrogram depicts sample clusters based on Pearson correlation coefficients, using the average-linkage method. Shading from blue to red indicates an increasing concentration of the corresponding metabolite

**Fig. 3:**
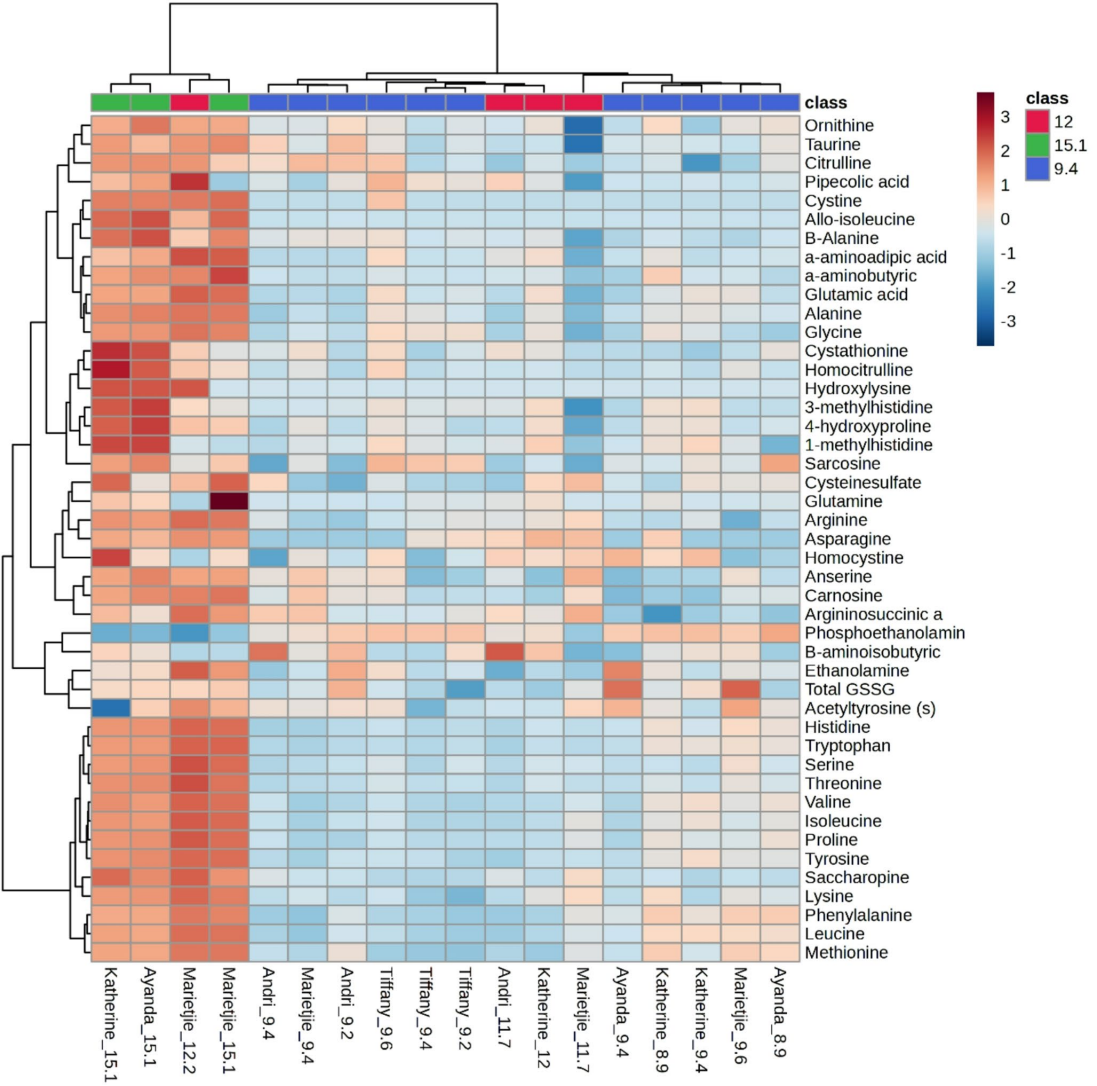
Heat-map visualisation and hierarchical clustering of amino acids and derivatives profiles of giraffe milk at 9.4, 12, and 15.1 months of lactation. The dendrogram depicts sample clusters based on Pearson correlation coefficients, using the average-linkage method. Shading from blue to red indicates an increasing concentration of the corresponding metabolite

A total of 37 organic acids and 45 amino acids (and their derivatives, including total glutathione) were measured (see Tables 1 and 2, respectively). PCA scatter plots of the organic acids of all the giraffe milk samples (Fig. 1A) illustrate that the first two principal components (PC1 and PC2) accounted for 53% and 24.7% of the total variation, respectively. PCA scatter plots of the amino acids and derivatives of all the giraffe milk samples (Fig. 1B) illustrate that the first two principal components (PC1 and PC2) accounted for 73.9% and 11.2% of the total variation, respectively. The samples were clustered into three groups by PCA, indicating good repeatability. There was some overlap among the groups, but the PCA scores indicated a trajectory of lactation progression from 9.4 to 15.1 months. In both organic acids and amino acids and derivatives, three samples at 12 months were more closely associated with samples at 9.4 months of lactation, while only one sample at 12 months overlapped with 15.1 months. Notably, all results at 9.4 months of lactation are clustered, indicating that all the giraffes were still in mid-lactation. The same applies to the milk of 15.1 months, which indicates a very late stage of lactation. At 12 months, uncertainty about the lactation stage widened the data spread. Heatmaps of organic acids (Fig. 2) and amino acids and derivatives (Fig. 3) were used to visualise the dynamic changes in metabolite abundance of the milk at 9.4, 12, and 15.1 months of lactation. Linear regression plots of all measured metabolites are given in the supplementary information.

The heat map of the organic acids (Fig. 2) can be divided into three main blocks based on the changes over lactation. The top block from 3-phenyllactic acid down to uracil occurred at high concentrations in the milk of three animals at 9.4 months of lactation and was reduced in the milk of 12 and 15.1 months of lactation. The middle block from 3-hydroxyisobutyric acid down to lactic acid indicates that these organic acids appeared at high concentration at 15.1 months of lactation. The block at the bottom from 3-hydroxy-3-methylbutyric acid to methylcitric acid indicates that the milk content of these organic acids was reduced at 15.1 months of lactation. The amino acid heat map (Fig. 3) showed that most were at low milk concentrations at 9.4 and 12 months of lactation and increased at 15.1 months of lactation.

**Table 1:** Organic acids (n = 37) detected in the milk from giraffe cows during lactation between months 9, 12, and 15

	Average concentration (µM) ± SD			R^2^	ANOVA	Effect size (g-value)		
Metabolites	9	12	15		(p-value)	9 vs 12	12 vs 15	9 vs 15
2,3-Pyridinecarboxylic acid	0.07 ± 0.05	1.53 ± 2.96	4.44 ± 1.04	0.453	**0.003**	**1.031**	**1.22**	**10.226**
2-Hydroxy-3-methylbutyric acid	1.03 ± 1.12	0.69 ± 0.74	1.61 ± 0.65	0.015	0.594	0.319	**1.294**	0.545
2-Hydroxybutyric acid	0.80 ± 0.62	74.85 ± 147.31	117.82 ± 77.81	0.35	**0.089**	**1.046**	0.346	**3.683**
2-Hydroxyglutaric	328.31 ± 130.27	329.89 ± 370.17	10.87 ± 4.51	0.008	0.121	0.007	**1.113**	**2.669**
2-Hydroxyisocaproic acid	4.86 ± 7.19	0.21 ± 0.30	0.59 ± 0.19	0.004	0.441	**0.739**	**1.467**	**0.651**
2-Ketoglutaric acid	50.65 ± 12.49	41.68 ± 29.89	1.80 ± 1.04	0.006	**0.005**	0.497	**1.722**	**4.283**
3-Hydroxy-3-methylglutaric acid	1.71 ± 1.20	1.71 ± 1.18	0.63 ± 0.22	0.125	0.452	0.003	**1.177**	**0.988**
3-Hydroxybutyric acid	7.08 ± 2.06	28.42 ± 29.42	96.17 ± 26.10	0.373	**<0.001**	**1.498**	**2.408**	**8.233**
3-Hydroxyhippuric acid	78.63 ± 26.66	75.21 ± 58.71	26.66 ± 10.11	0.298	0.162	0.094	**1.057**	**2.105**
3-Hydroxyisobutyric acid	0.36 ± 0.11	1.07 ± 1.19	3.96 ± 1.27	**0.654**	**<0.001**	**1.223**	**2.368**	**6.826**
3-Methyl-2-oxovaleric acid	0.49 ± 0.61	2.40 ± 3.62	10.57 ± 2.81	0.156	**<0.001**	**1.05**	**2.459**	**7.916**
3-Phenyllactic acid	1.63 ± 2.92	1.41 ± 2.74	0.36 ± 0.09	0.028	0.772	0.078	0.493	0.477
4-Hydroxybenzoic acid	0.85 ± 0.55	1.60 ± 2.23	1.06 ± 0.12	**0.836**	0.594	**0.633**	0.309	0.412
4-Hydroxyphenyllactic acid	0.38 ± 0.46	1.33 ± 2.57	1.11 ± 0.10	0.072	0.470	**0.731**	0.11	**1.731**
4-Methyl-2-oxovaleric acid	2.01 ± 3.05	3.21 ± 4.71	13.18 ± 3.85	**0.874**	**0.003**	0.345	**2.273**	**3.5**
4-Pyridoxic acid	20.03 ± 2.49	21.30 ± 11.31	22.52 ± 3.07	0.041	0.772	0.217	0.136	**0.96**
cis-Aconitic acid	179.42 ± 59.40	109.82 ± 68.89	22.78 ± 8.76	0.354	**0.006**	**1.128**	**1.622**	**2.882**
Citric acid	7047.70 ± 684.20	5655.36 ± 2769.43	1658.70 ± 145.19	**0.841**	**<0.001**	**0.954**	**1.861**	**8.589**
Fumaric acid	1.90 ± 1.48	2.43 ± 0.73	1.32 ± 0.64	0.046	0.594	0.396	**1.593**	0.423
Glyceric acid	0.52 ± 0.23	0.67 ± 0.33	0.60 ± 0.26	0.03	0.633	0.6	0.229	0.356
Hexanoylglycine	0.32 ± 0.03	0.30 ± 0.04	0.28 ± 0.03	0.016	0.352	**0.612**	0.424	**1.187**
Hippuric acid	53.79 ± 21.82	72.87 ± 11.59	65.77 ± 15.44	0.005	0.363	**0.958**	0.536	0.574
Isocitric acid	55.74 ± 7.94	40.50 ± 22.92	12.23 ± 0.25	**0.744**	**0.001**	**1.17**	**1.592**	**6.003**
Lactic acid	109.06 ± 53.75	200.46 ± 285.11	723.61 ± 82.38	**0.661**	**<0.001**	**0.631**	**2.306**	**10.331**
Malic acid	373.37 ± 268.09	495.70 ± 91.49	294.87 ± 72.91	0.423	0.594	0.511	**2.375**	0.318
Malonic acid	0.81 ± 0.41	0.95 ± 0.60	3.82 ± 0.48	0.067	**<0.001**	0.298	**5.195**	**7.119**
Methylcitric acid Peak 1	3.00 ± 0.70	2.60 ± 1.76	0.59 ± 0.40	0.046	**0.015**	0.385	**1.451**	**3.668**
Methylcitric acid Peak 2	5.20 ± 0.98	4.64 ± 2.86	1.25 ± 0.27	**0.708**	**0.010**	0.346	**1.524**	**4.403**
Orotic acid	84.37 ± 22.60	60.35 ± 3.19	14.11 ± 2.47	0.155	**0.001**	**1.208**	**15.819**	**3.401**
Propionylglycine	0.78 ± 0.28	1.01 ± 0.95	2.27 ± 0.82	0.059	**0.010**	0.442	**1.392**	**3.505**
Pyroglutamic acid	41.89 ± 20.96	868.90 ± 1549.16	1328.58 ± 793.58	**0.697**	**0.033**	**1.111**	0.353	**3.965**
Pyruvic acid	5.05 ± 3.18	1.94 ± 1.19	5.04 ± 4.81	0.009	0.376	**1.092**	**0.976**	0.003
Sebacic acid	0.72 ± 0.10	0.97 ± 0.49	1.91 ± 0.43	**0.766**	**<0.001**	**0.969**	**2.007**	**6.01**
Suberic acid	0.87 ± 0.09	0.95 ± 0.25	1.35 ± 0.08	0.019	**0.001**	0.547	**2.001**	**5.29**
Succinic acid	229.20 ± 133.86	134.77 ± 20.13	110.19 ± 32.30	0.193	0.285	**0.802**	**0.956**	**0.968**
trans-Aconitic acid	30.75 ± 47.58	5.11 ± 5.49	1.77 ± 0.90	0.06	0.478	**0.613**	**0.777**	**0.667**
Tricarballylic acid	0.43 ± 0.92	0.20 ± 0.37	0.09 ± 0.12	0.031	0.772	0.272	0.4	0.408
Uracil	85.85 ± 62.48	20.55 ± 39.85	11.94 ± 8.44	0.071	0.108	**1.125**	0.275	**1.294**

Data reported as average concentration (µM) ± standard deviation. Organic acids that exhibit strong linear correlation (R^2^ > 0.6), statistical significance (ANOVA FDR p-value < 0.05, and practical significance (effect size g-value > 0.8) are given in bold

**Table 2:** Amino acids (and their derivatives, including total glutathione; *n* = 45) detected in the milk from giraffe cows during lactation between months 9, 12, and 15

	Average concentration (µM) ± SD			*R* ^2^	ANOVA	Effect size (g-value)		
Metabolites	9	12	15		(*p*-value)	9 vs. 12	12 vs. 15	9 vs. 15
1-Methylhistidine	1.40 ± 0.66	1.36 ± 1.18	14.26 ± 7.86	**0.812**	0.129	0.126	**1.707**	**2.61**
2-Aminoadipic acid	0.22 ± 0.04	0.28 ± 0.37	0.45 ± 0.15	**0.646**	**0.034**	**1.036**	0.27	**3.935**
2-aminobutyric acid	1.18 ± 1.33	1.47 ± 10.80	19.63 ± 30.26	**0.644**	**0.003**	**0.941**	**1.318**	**2.621**
3-Aminoisobutyric acid	0.34 ± 0.07	0.35 ± 0.13	0.36 ± 0.04	0.066	0.945	0.289	0.257	0.023
3-Methylhistidine	1.12 ± 0.32	1.53 ± 0.74	7.09 ± 3.93	0.14	**0.014**	0.286	**1.747**	**2.814**
4-Hydroxyproline	1.59 ± 0.49	1.97 ± 1.57	11.60 ± 6.29	0.378	**0.004**	0.543	**1.979**	**3.339**
Acetyltyrosine	0.32 ± 0.07	0.33 ± 0.10	0.38 ± 0.14	0.22	0.754	0.364	0.316	0.118
Alanine	63.80 ± 28.63	61.96 ± 416.03	729.61 ± 96.46	0.041	**0.005**	**0.92**	**1.4**	**13.635**
Allo-isoleucine	0.46 ± 0.01	0.46 ± 0.36	2.31 ± 2.88	0.012	**< 0.001**	**1.011**	**1.802**	**2.974**
Anserine	0.08 ± 0.11	0.35 ± 0.33	0.74 ± 0.23	0.198	**0.017**	**1.245**	**1.637**	**5.272**
Arginine	5.82 ± 3.55	21.77 ± 124.90	128.69 ± 53.87	**0.867**	**0.001**	**1.232**	0.663	**6.387**
Argininosuccinic acid	0.21 ± 0.31	1.01 ± 1.63	1.14 ± 0.82	0.162	0.054	**1.559**	0.256	**2.153**
Asparagine	0 ± 0.29	3.74 ± 17.97	11.53 ± 9.60	**0.941**	**0.001**	**1.336**	0.095	**3.305**
Carnosine	0.71 ± 0.60	0.94 ± 3.27	6.38 ± 2.12	**0.719**	**0.007**	**0.977**	**1.424**	**5.576**
Citrulline	1.36 ± 1.89	0.94 ± 4.71	9.81 ± 3.64	0.462	0.144	0.261	**1.185**	**2.567**
Cystathionine	0.31 ± 0.03	0.36 ± 0.04	0.59 ± 0.16	0.099	**0.005**	**0.934**	**1.579**	**2.796**
Cysteine sulfate	0.60 ± 0.07	0.73 ± 0.12	0.96 ± 0.19	0.201	**0.014**	**1.176**	**1.161**	**2.743**
Cystine	0 ± 0.06	0 ± 4.95	8.08 ± 5.80	0.014	**0.002**	**1.032**	**1.586**	**4.591**
Ethanolamine	15.47 ± 26.28	8.86 ± 69.13	27.75 ± 29.18	0.05	0.496	0.387	0.014	0.613
Glutamic acid	116.75 ± 64.20	169.87 ± 735.43	700.25 ± 381.61	**0.959**	**0.015**	**0.987**	0.667	**4.603**
Glutamine	60.68 ± 2.53	69.86 ± 124.72	86.30 ± 76.03	0.022	0.057	**1.121**	0.02	**2.09**
Glycine	74.55 ± 51.69	84.19 ± 413.33	564.95 ± 88.19	0.214	**0.016**	**0.857**	**1.049**	**8.684**
Histidine	7.61 ± 8.65	7.61 ± 156.98	143.48 ± 74.40	**0.854**	**0.005**	**0.979**	0.751	**5.485**
Homocitrulline	0.55 ± 0.01	0.55 ± 0.01	0.59 ± 0.02	0.154	**0.002**	0.403	**2.035**	**3.333**
Homocystine	0.37 ± 0.04	0.39 ± 0.02	0.39 ± 0.03	0.011	0.441	0.173	**0.935**	0.8
Hydroxylysine	0 ± 0	0 ± 0.16	0.33 ± 0.20	0.519	**0.021**	**1.041**	**0.826**	**2.819**
Isoleucine	12.87 ± 9.39	18.12 ± 394.44	269.36 ± 201.15	**0.972**	**0.004**	**1.033**	0.483	**4.31**
Leucine	34.86 ± 53.51	32.46 ± 718.41	540.33 ± 466.35	**0.986**	**0.020**	**0.933**	0.626	**3.659**
Lysine	20.06 ± 24.58	68.53 ± 492.78	433.77 ± 149.10	0.086	**0.021**	**1.139**	0.532	**7.398**
Methionine	2.80 ± 10.15	3.63 ± 120.75	81.32 ± 92.59	0.328	**0.026**	**0.93**	0.632	**3.194**
Ornithine	14.30 ± 6.71	15.42 ± 27.10	60.05 ± 31.38	**0.71**	**0.034**	0.532	**1.828**	**4.255**
Phenylalanine	18.87 ± 31.66	16.09 ± 233.89	189.09 ± 117.96	0.338	**0.037**	**0.857**	0.639	**3.995**
Phosphoethanolamine	330.40 ± 70.90	140.33 ± 86.92	66.86 ± 10.99	**0.683**	**< 0.001**	**2.418**	**1.023**	**3.859**
Pipecolic acid	0.40 ± 0.15	0.55 ± 0.84	0.78 ± 0.37	0.551	0.644	**0.893**	0.214	**1.127**
Proline	25.39 ± 22.95	34.49 ± 1281.38	820.28 ± 501.79	**0.843**	**0.004**	**1.035**	0.387	**5.051**
Saccharopine	0.25 ± 0.02	0.32 ± 0.18	0.54 ± 0.05	0.402	**0.001**	**1.488**	**1.227**	**11.429**
Sarcosine	0.65 ± 0.32	0.49 ± 0.17	1.36 ± 0.30	0.085	**0.032**	**0.899**	**3.551**	**1.764**
Serine	25.43 ± 14.46	31.37 ± 393.46	302.41 ± 151.72	**0.969**	**0.004**	**1.03**	0.461	**5.407**
Taurine	72.08 ± 25.23	59.98 ± 75.98	185.07 ± 34.81	**0.74**	**0.038**	0.05	**1.539**	**3.654**
Threonine	12.64 ± 4.94	14.28 ± 212.95	174.72 ± 50.74	0.509	**0.004**	**1.021**	0.472	**8.671**
Total glutathione	0.56 ± 0.37	0.53 ± 0.15	0.75 ± 0.05	0.007	**0.330**	0.431	**1.681**	0.176
Tryptophan	4.06 ± 3.52	3.96 ± 62.10	54.34 ± 40.02	0.429	**0.006**	**0.951**	0.768	**4.219**
Tyrosine	12.60 ± 10.87	11.31 ± 254.04	266.45 ± 115.10	**0.986**	**0.005**	**0.98**	**0.842**	**6.177**
Valine	57.56 ± 46.51	57.83 ± 772.17	796.46 ± 322.65	0.217	**0.005**	**0.978**	0.752	**6.071**
β-Alanine	2.51 ± 0.51	2.74 ± 1.42	9.39 ± 1.83	0.042	**< 0.001**	0.206	**4.287**	**7.994**

Data reported as average concentration (µM) ± standard deviation. Amino acids that exhibited strong linear correlation (R^2^ > 0.6), statistical significance (ANOVA FDR p-value < 0.05), and practical significance (effect size g-value > 0.8) are given in bold

### Significantly different metabolites

Statistically different metabolites were determined by ANOVA (FDR p-value < 0.05), supported by a strong linear trend (R^2^ > 0.6) and practical significance (effect size g-value > 0.8). The major components of the giraffe milk metabolome that changed significantly from mid (9 to 12 months) to late (15 months) lactation consisted of 19 organic acids and 14 amino acids. For the amino acids, phosphoethanolamine decreased, and the other 13 significant amino acids increased. Between months 9.4 and 15.1, 12 organic acids increased, and 7 decreased. Due to the overlap between groups 9.4 and 12, and 12 and 15.1 in the PCA, as well as the small number of significantly different metabolites between them, it was decided to focus all subsequent comparisons on the 9.4 and 15.1 months of lactation for the discussion.

## Discussion

As the introduction above indicates, literature on giraffes is scarce, particularly regarding longitudinal changes during lactation. Almost all hypotheses of the current study are based on the literature on cows and humans, which might be a limitation of the work. The PCA clustered the organic and amino acids (Fig. 1), indicating good repeatability. Although there was some overlap among the groups, a trajectory of lactation progression from 9.4 to 15.1 months was observed. This confirms that advancing lactation caused notable alterations in the organic acids, as well as in the amino acids and their derivatives, in the milk metabolic profile. The observation that three samples at 12 months of lactation were more closely associated with samples at 9.4 months of lactation, while only one sample at 12 months overlapped with 15.1 months, might be evidence of individual differences in mammary involution timing rather than a uniform transition.

The results from this targeted LC-MS/MS metabolomics study support the results from our untargeted ^1^H-NMR metabolomics approach (Osthoff et al., 2026). Here, 37 organic acids and 45 amino acids (and their derivatives, including total glutathione) were identified in giraffe milk, representing a vast increase over the 19 and 14 identified in our previous report (Osthoff et al., 2026). Using stepwise analysis, we provide a consolidated biological interpretation of the changes in metabolites and the metabolic pathways, as detailed in this discussion.

The dynamic changes of the organic acids (Fig. 2) and amino acids from 9.4 to 12 and finally to 15.1 months, together with the statistical data (Tables 1 and 2) were used derive interpretations of metabolic pathways.

### Reduced oxidative and biosynthetic metabolism through the Krebs cycle

**Fig. 4:**
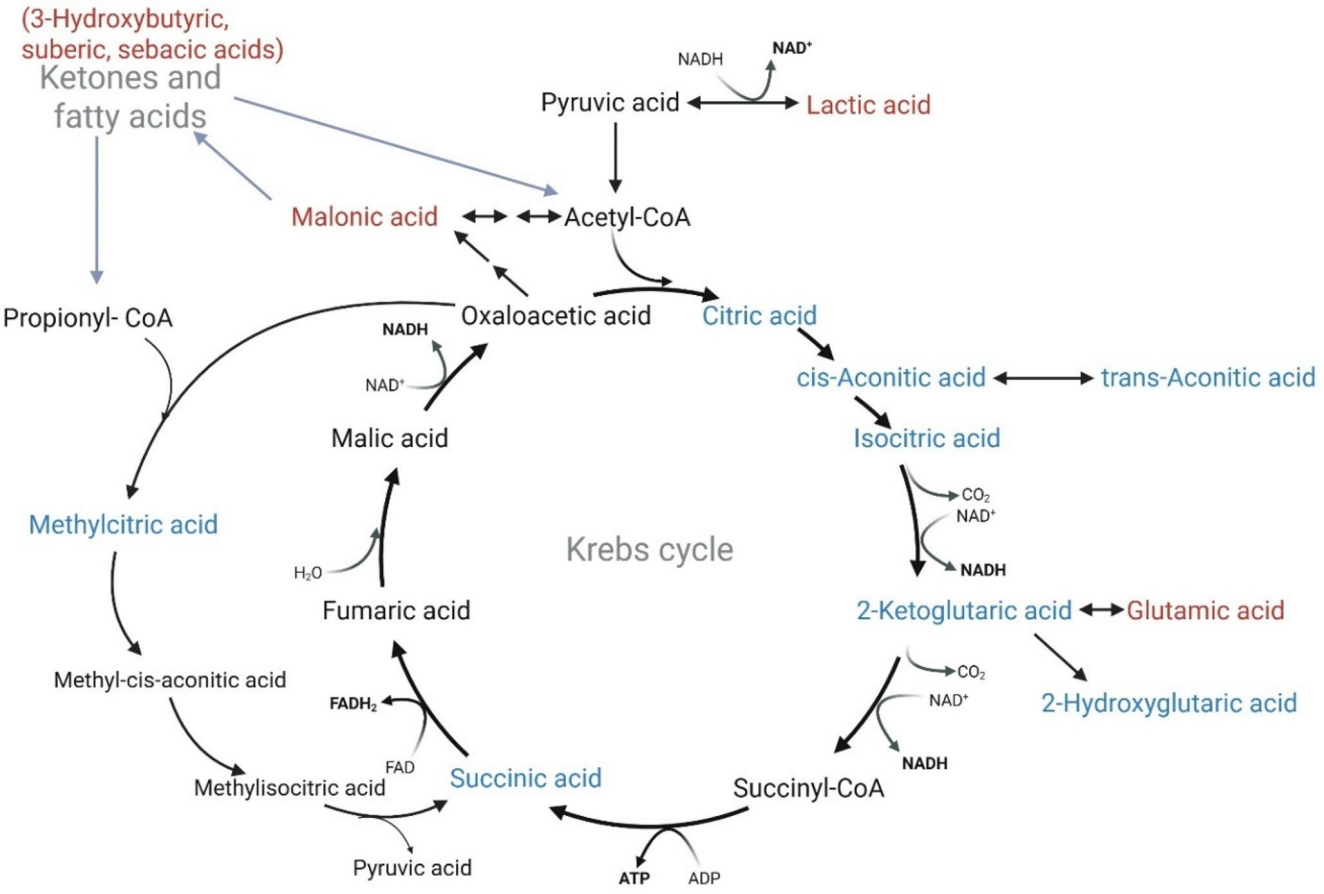
Metabolic pathways linked to the Krebs cycle showing change (blue = decrease; red = increase) in giraffe milk from mid to late lactation. The overall trend across all Krebs cycle intermediates and associated organic acids is decreased availability in giraffe milk at late lactation. However, there is an increase in lactic, glutamic, and malonic acids, as well as increased ketones (3-hydroxybutyric acid) and fatty acids (suberic acid and sebacic acid)

Figure 4 prominently illustrates a decrease of Krebs cycle intermediates (citric, cis-aconitic, isocitric, 2-ketoglutaric, and succinic acids) and associated organic acids (trans-citric, 2-hydroxyglutaric, and methylcitric acids) as giraffe milk transitions from mid to late lactation. This widespread decrease suggests reduced oxidative and biosynthetic metabolism through the Krebs cycle (Korla & Mitra, 2014) in the mammary gland, consistent with declining anabolic demand and milk volume production as lactation progresses. Reduced citrate availability further implies diminished provision of citrate for lactose-associated osmotic regulation (Hamon et al., 2025), a process known to peak earlier in lactation.

In contrast, lactic acid and malonic acid increased at late lactation, indicating a metabolic shift toward glycolytic and regulatory adaptations. Elevated lactic acid suggests increased reliance on anaerobic glycolysis or altered redox balance (NADH/NAD⁺), potentially reflecting reduced mitochondrial activity in mammary epithelial cells (Choi et al., 2025). Increased malonic acid, a known inhibitor of succinate dehydrogenase (Valls-Lacalle et al., 2016), may further contribute to the suppression of the Krebs cycle throughput. Moreover, increased malonic acid can be linked to increased ketones and fatty acids (3-hydroxybutyric, suberic, and sebacic acids) (Foster, 2012), indicating enhanced fatty acid β-oxidation and ω-oxidation, likely reflecting greater mobilisation of maternal lipid reserves in late lactation. Overall, the pathway-level changes reflect a metabolic transition from high biosynthetic and oxidative activity in mid lactation to a more catabolic, energy-efficient, and lipid-supported metabolic state in late lactation, aligning mammary metabolism with evolving maternal physiological demands.

### Adaptive nitrogen redistribution and redox buffering

**Fig. 5:**
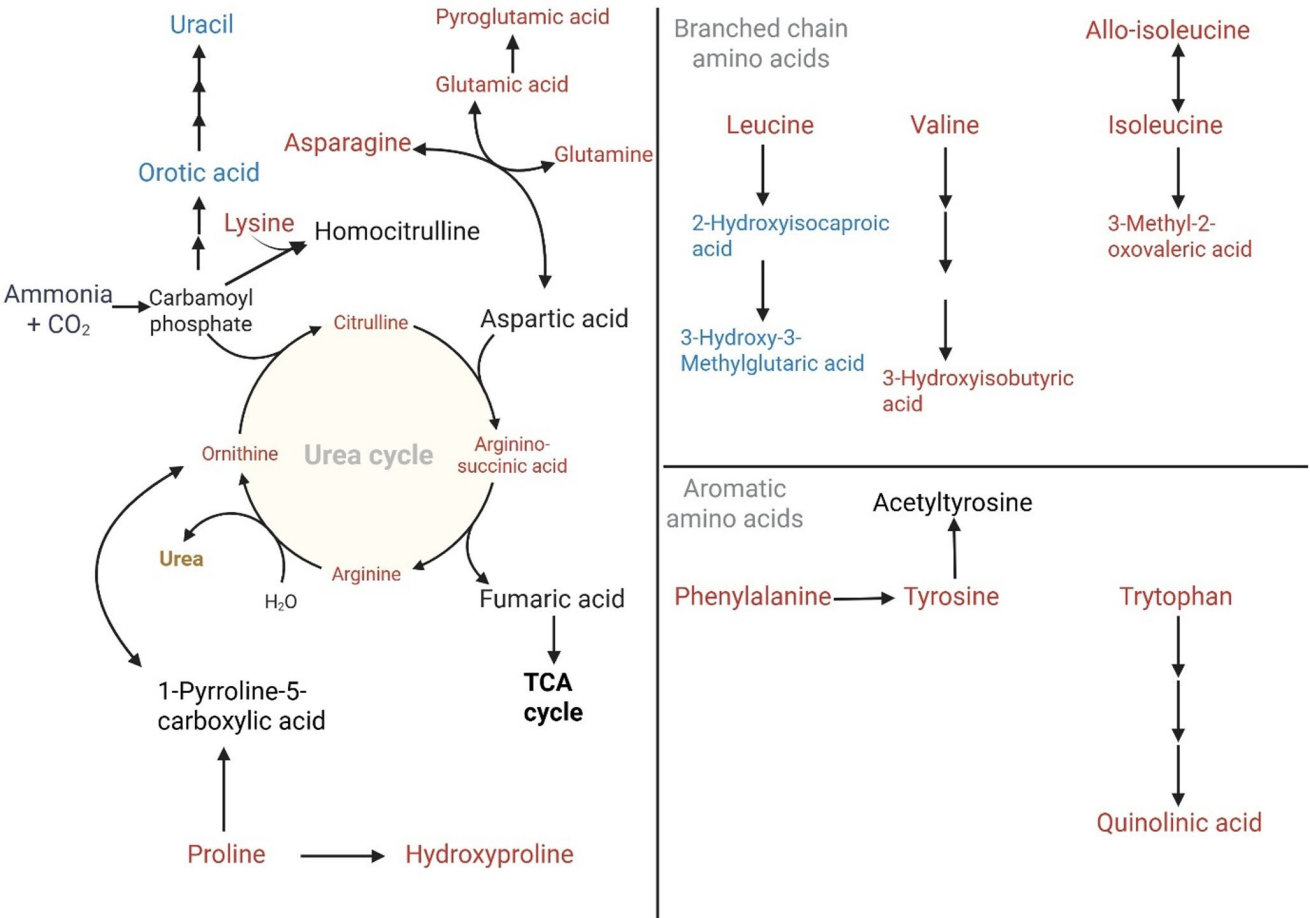
Urea cycle metabolites showing change (blue = decrease; red = increase) in giraffe milk from mid to late lactation. The overall trend across all urea-cycle metabolites is increased availability in late lactation. Consequently, there are decreased pyrimidine catabolites (orotic acid and uracil)

The dominant feature shown in Fig. 5 is the increase of urea cycle intermediates (arginine, ornithine, citrulline, and argininosuccinate), as well as other linked amino acids (lysine, proline, asparagine, and glutamic acid). The trend shown in Fig. 5 suggests enhanced nitrogen handling, reduced anabolic demand, and increased protein turnover in the mammary gland during late lactation. The decrease in Krebs cycle intermediates (Fig. 4) also suggests reduced metabolism of argininosuccinic acid to fumaric acid (another Krebs cycle intermediate), indicating increased nitrogen excretion as urea as lactation comes to an end.

**Fig. 6:**
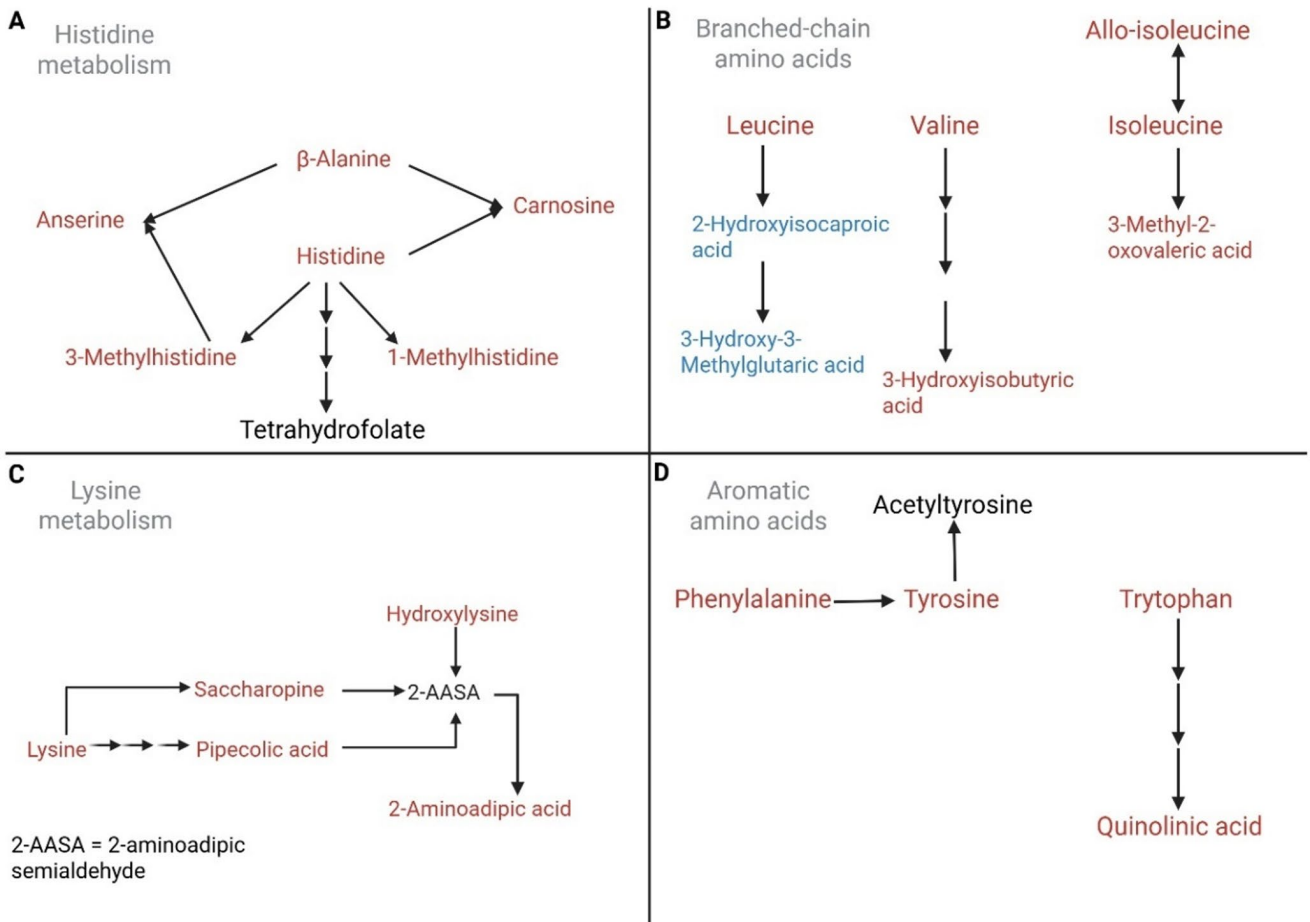
Metabolic pathways showing changes in amino acids and associated downstream organic acid catabolites (blue = decrease; red = increase). **A** – Histidine metabolism. **B** – Branched-chain amino acids. **C** – Lysine metabolism. **D** – Aromatic amino acids

At the same time, elevated glutamic acid, glutamine, pyroglutamic acid, and asparagine indicate intensified glutamine–glutamate cycling, which reflects adaptive nitrogen redistribution and redox buffering (Geigenberger & Fernie, 2014). Increased pyroglutamic acid may also reflect heightened γ-glutamyl cycle activity, associated with antioxidant and amino acid transport processes (Emmett, 2014; Orlowski & Meister, 1970). In addition, decreased levels of uracil and orotic acid from mid to late lactation suggest decreased *de novo* pyrimidine synthesis, consistent with reduced nucleic acid synthesis as lactation progresses. Collectively, these changes indicate a shift from anabolic protein and nucleotide synthesis toward nitrogen recycling, amino acid remodelling, and metabolic efficiency in late lactation, aligning mammary metabolism with declining milk yield and reduced offspring requirements.

### Amino acid perturbation

**Fig. 7:**
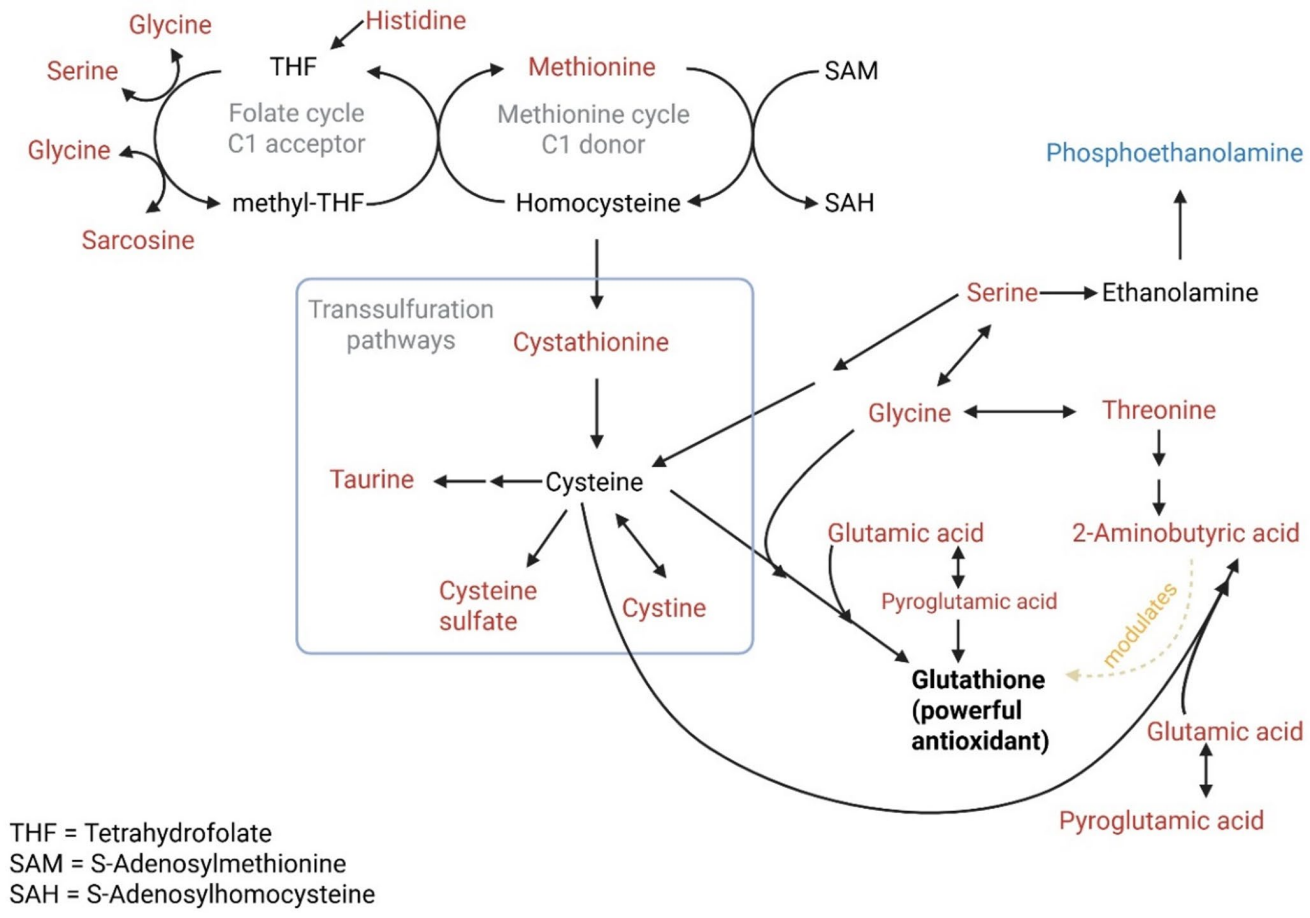
Metabolic pathways showing changes in one-carbon metabolism (folate and methionine cycles), transsulfuration pathways, and associated metabolites linked to glutathione synthesis (blue = decrease; red = increase)

Figure 6 shows an overall increase of amino acids as the giraffe milk transitions to late lactation. Increased histidine (Fig. 6A) and its methylated forms (1-methylhistidine and 3-methylhistidine) suggest increased protein degradation (Elia et al., 1981) in the mammary glands toward late lactation. Anserine and carnosine act as antioxidants and pH buffers (preventing acidosis) (Kohen et al., 1988; Suzuki et al., 2006), with β-alanine being the rate-limiting precursor of the synthesis (Trexler et al., 2015). Hence, an overall increase of these metabolites together indicates an increased ergogenic effect. Increased histidine is also associated with increased tetrahydrofolate and one-carbon metabolism (Fig. 7) (Brosnan & Brosnan, 2020).

Branched-chain amino acids (BCAAs; leucine, valine, and isoleucine – Fig. 6B) were increased, alongside catabolic intermediates for valine (3-hydroxyisobutyric acid) and isoleucine (3-methyl-2-oxovaleric acid), indicating altered BCAA utilisation. However, leucine was increased, but its catabolic intermediates (2-hydroxyisocarpoic acid and 3-hydroxy-3-methylglutaric acid) were decreased, possibly reflecting that leucine is needed alone for some purpose.

Increased lysine metabolism (Fig. 6C) shows increased levels of lysine catabolites saccharopine, pipecolic acid, and 2-aminoadipic acid. Increased lysine catabolism is often associated with mitochondrial disorders (Zhou et al., 2019), in which there is decreased availability of energy metabolites and an altered redox balance (increased NADH: NAD^+^). Hence, the presence of these lysine metabolites is likely linked to stress adaptation as the mother approaches the end of lactation, as well as to post-translational modifications that regulate protein function and cellular signalling (Eftekhari et al., 2025).

An overall increase in aromatic amino acids (phenylalanine, tyrosine, tryptophan) and the tryptophan downstream catabolite quinolinic acid (Fig. 6D) suggests enhanced aromatic amino acid turnover and potential modulation of immune and neuroactive metabolite supply (Shin & Bang, 2025).

### One-carbon, transsulfuration, and glutathione metabolism

Figure 7 shows that increased histidine catabolism feeds directly into the folate-dependent one-carbon (C1) metabolism, with downstream consequences for methylation capacity, sulfur amino acid metabolism, and antioxidant defence. Histidine degradation transfers its formimino group to tetrahydrofolate (THF), which is subsequently converted to methyl-THF. The observed increases in glycine, serine, and sarcosine support the hypothesis of increased one-carbon metabolism during late lactation. Network propagation from the folate cycle into the methionine cycle is supported by increased levels of methionine and transsulfuration intermediates, along with diversion of homocysteine toward cystathionine and cysteine biosynthesis. Increased one-carbon metabolism can also be linked to increased methylation reactions, including epigenetic regulation (Friso et al., 2017; Khot et al., 2015) and phospholipid remodelling (e.g., serine → ethanolamine → phosphoethanolamine).

Increased conversion of homocysteine to cystathionine indicates activation of transsulfuration pathways, diverting sulfur metabolism away from methionine recycling toward the synthesis of cysteine, taurine, and glutathione. Elevated cysteine, cystine, taurine, and cysteine sulfate are consistent with a metabolic strategy favouring redox buffering and cytoprotection over protein synthesis (Zhu et al., 2019).

The concomitant accumulation of glutamate and pyroglutamate further supports enhanced γ-glutamyl cycle activity, with the hypothesised increase in glutathione turnover. Elevated 2-aminobutyric acid, a by-product of transsulfuration and modulator of glutathione synthesis (Irino et al., 2016), serves as a marker of increased glutathione demand and oxidative stress adaptation. To test this specific hypothesis, we directly measured the total glutathione content (oxidised + reduced glutathione) of the giraffe milk, but did not find any linear trend (R^2^ = 0.007); instead, there was only some practical significance (g-value = 1.68) as total glutathione increased from 0.53 ± 0.15 at 12 months to 0.75 ± 0.05 at end of lactation (15 months). Hence, our study found insufficient evidence to support our hypothesis that glutathione levels increase significantly as the giraffe approaches the end of lactation. Future studies should examine other antioxidant systems and their redox-buffering capacity in giraffe milk.

### Biochemical results in the context of the agricultural sector

In the commercial milk production sector, the management of a healthy dry-out and dry period is important to ensure optimal milk gland function during subsequent lactation (Odensten, 2007; Hurley & Loor, 2011). In giraffes, this does not apply because giraffe females often become pregnant before a calf is weaned (Bercovitch & Berry, 2013). A limitation of this study was that our initial research was not planned to study the dry-out period of lactation, and the exact lactation stage could not be determined. Nevertheless, a description of the metabolomic changes from energy production to redox buffering and cytoprotection was possible.

Since the dynamic changes of proximate (Osthoff et al. 2025b) and metabolome composition (Osthoff et al., 2026) of giraffe milk from mid lactation to dry-out have now been extensively described, it would also be appropriate to map the complete lactation from colostrum, throughout early lactation, to mature milk, and, eventually, involution. Other investigation methods, such as proteomics and lipidomics, should also be employed.

## Conclusions

This work concluded that involution, or the dry-out, of the giraffe cow occurs at approximately 15 months of lactation, although it may commence at approximately 12 months, as previously reported by Osthoff et al., 2026. At the specific stage at which involution was captured in our study, the involution was accompanied by a replacement of the role of metabolites from energy production to antioxidant, redox buffering and cytoprotective functions.

Decreased Krebs cycle intermediates and a lower citrate content lead to lower lactose levels, indicating a reduced role for osmotic regulation. Lower uracil and orotic acid indicate decreased pyrimidine synthesis and eventual nucleotide synthesis. The increased amino acid content is no longer devoted to protein synthesis but redirected to other functions, specifically as antioxidants, redox buffers, and cytoprotective metabolites. Increased contents of histidine, serine and methionine promote protein degradation and one-carbon metabolism. Increased lysine catabolites lead to decreased energy metabolites and stress adaptation, while increased levels of aromatic amino acids modulate immune and neuroactive metabolite supply.

In summary, there is, undoubtedly, a clearly discernible change in the milk metabolome between mid and late lactation. Further research is needed to confirm these results and to compare the giraffe milk metabolome with those of other ruminants.

## Supplementary Information

Below is the link to the electronic supplementary material.

Supplementary Material 1 (Linear regression plots of the measured metabolites are given in the supplementary information.)

Supplementary Material 2 (Linear regression plots of the measured metabolites are given in the supplementary information.)

## Data Availability

The data is made available as supplementary information.
