# Supplementary material for: Targeted metabolomics of organic and amino acids in giraffe milk during mid- to late-lactation
Source: Metabolomics. 2026 May 16;22(3):76. doi: 10.1007/s11306-026-02455-z (PMC13179917; doi:10.1007/s11306-026-02455-z)
Supplement: Supplementary file 1 — Supplementary Material 1 (Linear regression plots of the measured metabolites are given in the supplementary information.) [file 11306_2026_2455_MOESM1_ESM.docx]

Targeted LC-MS/MS metabolomics of giraffe milk during mid- to late-lactation

Running title: Giraffe milk metabolome during involution

Osthoff, G.*^a^, Mason, S.^b^, Davoren, E.^c^ Deacon, F.^d^

^a^ Department of Microbiology and Biochemistry, University of the Free State, Bloemfontein, South Africa.

^b^ Biomedical and Molecular Metabolism Research (BioMMet), Faculty of Natural and Agricultural Sciences, North-West University, Potchefstroom, South Africa.

^c.^ Centre for Human Metabolomics, Desmond Tutu School of Medicine, Faculty of Health Science, North-West University, Potchefstroom, South Africa.

^d^ Department of Animal, Wildlife and Grassland Sciences, University of the Free State, Bloemfontein, 9300, South Africa.

**Figure S1.** Linear regression plots of the 37 measured organic acids.

**Figure S2.** Linear regression plots of the 45 measured amino acids (and their derivatives, including total glutathione).
